# Oral Erythritol Reduces Energy Intake during a Subsequent *ad libitum* Test Meal: A Randomized, Controlled, Crossover Trial in Healthy Humans

**DOI:** 10.3390/nu14193918

**Published:** 2022-09-21

**Authors:** Fabienne Teysseire, Emilie Flad, Valentine Bordier, Aleksandra Budzinska, Nathalie Weltens, Jens F. Rehfeld, Christoph Beglinger, Lukas Van Oudenhove, Bettina K. Wölnerhanssen, Anne Christin Meyer-Gerspach

**Affiliations:** 1 St. Clara Research Ltd. at St. Claraspital, 4002 Basel, Switzerland; 2 Faculty of Medicine, University of Basel, 4001 Basel, Switzerland; 3 Translational Research Center for Gastrointestinal Disorders, Laboratory for Brain-Gut Axis Studies, Department of Chronic Diseases and Metabolism, Catholic University of Leuven, 3000 Leuven, Belgium; 4 Leuven Brain Institute, Catholic University of Leuven, 3000 Leuven, Belgium; 5 Department of Clinical Biochemistry, Rigshospitalet, University of Copenhagen, 1172 Copenhagen, Denmark; 6 Cognitive and Affective Neuroscience Lab, Department of Psychological and Brain Sciences, Dartmouth College, Hanover, NH 03755, USA

**Keywords:** energy intake, erythritol, sucrose, sucralose, gastrointestinal satiation hormone, cholecystokinin, healthy participants, low-caloric sweeteners

## Abstract

The impact of oral erythritol on subsequent energy intake is unknown. The aim was to assess the effect of oral erythritol compared to sucrose, sucralose, or tap water on energy intake during a subsequent *ad libitum* test meal and to examine the release of cholecystokinin (CCK) in response to these substances. In this randomized, crossover trial, 20 healthy volunteers received 50 g erythritol, 33.5 g sucrose, or 0.0558 g sucralose dissolved in tap water, or tap water as an oral preload in four different sessions. Fifteen minutes later, a test meal was served and energy intake was assessed. At set time points, blood samples were collected to quantify CCK concentrations. The energy intake (*ad libitum* test meal) was significantly lower after erythritol compared to sucrose, sucralose, or tap water (*p* < 0.05). Before the start of the *ad libitum* test meal, erythritol led to a significant increase in CCK compared to sucrose, sucralose, or tap water (*p* < 0.001). Oral erythritol given alone induced the release of CCK before the start of the *ad libitum* test meal and reduced subsequent energy intake compared to sucrose, sucralose, or tap water. These properties make erythritol a useful sugar alternative.

## 1. Introduction

Around 30% of people worldwide have overweight or obesity [1], representing a major susceptibility for metabolic diseases such as type 2 diabetes mellitus (T2DM). Several studies have reported that the number of sugar-sweetened beverages (SSBs) consumed correlates with the body mass index (BMI) as well as T2DM [2,3,4]. Consequently, the World Health Organization (WHO) recommends reducing sugar consumption [5].

Substituting sugar with artificial low-caloric sweeteners (LCS), such as sucralose or aspartame, might be a strategy for reducing calories while preserving sweet taste. However, the use of artificial LCS has not achieved the expected results. This may be in part because artificial LCS, given in isolation, have no effect on the release of gastrointestinal (GI) hormones and gastric emptying, possibly explaining the lack of effects on satiety, fullness, and digestive mechanisms [6,7,8,9,10]. Moreover, a review of in vitro and in vivo animal studies reported the negative effects of artificial LCS on glucose homeostasis [11], and an observational study in postmenopausal women indicated that artificial LCS consumption might increase the risk of developing T2DM [12]. However, the results of reviews and meta-analyses investigating the relationship between artificial LCS and glucose homeostasis are controversial [11,13,14,15]. These discrepancies presumably arise from differences in chemical properties and the biological fate of the artificial LCS, as well as the intake of artificial LCS in isolation or in a combination with other nutrients [11,16]. Dalenberg et al. [17] observed that the combination of sucralose with carbohydrates (e.g., in a meal) alters insulin sensitivity and glucose tolerance possibly because of the up-regulation of glucose transporters observed in animal models in response to artificial LCS consumption [18,19].

Because of the controversial data on artificial LCS in humans, low-caloric bulk sweeteners are attractive alternatives. Erythritol, a sugar alcohol with zero calories and a relative sweetness of 60–70% relative to sucrose, and according to some references even up to 80% [20,21,22], is associated with several positive physiological effects. In humans, acute ingestion of erythritol leads to an increase in GI satiation hormones (cholecystokinin (CCK), glucagon-like peptide 1 (GLP-1), and peptide tyrosine tyrosine (PYY)), slows down gastric emptying without affecting glucose and insulin concentrations, as well as blood lipids [23,24,25,26]. Regarding the effects on energy intake following erythritol consumption, the one human study to investigate partial sucrose replacement by erythritol in a test breakfast reported similar levels of energy intake, GI satiation hormone release (GLP-1 and PYY), and satiety between erythritol and sucrose [27]. However, no pure erythritol was used, and hence the effect of oral erythritol on subsequent energy intake is still unknown.

The primary objective of this study was to investigate the effect of oral erythritol compared to sucrose, sucralose, or tap water on energy intake during a subsequent *ad libitum* test meal in healthy participants. As a secondary aim, we examined the release of the GI satiation hormone CCK, glycemic control, and appetite-related sensations in response to these substances.

We hypothesized that erythritol will lead to a similar subsequent energy intake during the *ad libitum* test meal as sucrose and to a lower energy intake compared to sucralose or tap water.

## 2. Participants and Methods

### 2.1. Participants

Twenty-six healthy participants were recruited via advertisement at the University of Basel and were eligible for the study when fulfilling all of the following inclusion criteria: age between 18–55 years, BMI of 19.0–24.9 kg/m^2^, and normal eating habits (eating breakfast, no diets, no dietary changes, no vegetarians/vegans, no intolerances/allergies). The exclusion criteria were working night shifts, fructose intolerance, substance and alcohol abuse, acute or chronic infections, chronic medical illness, illnesses affecting the GI system, pre-existing consumption of erythritol and/or sucralose more than once a week, pregnancy, and participation in another study with an investigational drug within 30 days preceding and during the present study.

### 2.2. Ethical Approval

The trial was approved by the regional Ethics Committee of Basel, Switzerland (Ethikkommission Nordwest-und Zentralschweiz (EKNZ): 2020-02847) and conducted in compliance with the current version of the Declaration of Helsinki and national legal and regulatory requirements. Each participant gave written informed consent for trial participation. The study was registered at ClinicalTrials.gov (NCT04713137).

### 2.3. Study Design and Procedure

The study utilized a randomized, placebo-controlled, double-blind, four-way crossover design. Participants attended four test sessions at least 1 week apart. All sessions were conducted between February and June 2021. During the 24 h preceding each visit, participants were asked to refrain from physical activities, alcohol, and caffeine. After a standardized dinner (consisting of pasta, a chocolate bar, and a soup; total 783 kcal), participants had to do an overnight fast from 08:00 PM until admission to the St. Clara Research Ltd., Basel, Switzerland, the next morning. All studies started at 08:00 AM to account for the circadian rhythm of GI satiation hormones. A cannula was inserted into a forearm vein for blood collection. After taking a fasting blood sample (*t* = −16 min), participants received one of the equisweet preloads (at *t* = −15 min) in a randomized order and had 2 min to consume it:

− 50 g erythritol;
− 33.5 g sucrose;
− 0.0558 g sucralose;
− 300 mL tap water.

Erythritol, sucrose, and sucralose were dissolved in 300 mL tap water. The doses of erythritol, sucrose, and sucralose were matched regarding sweetness. Based on previous studies, 50 g erythritol releases GI satiation hormones without side effects and accounts to a relative sweetness of 67% of sucrose (33.5 g, a typical sweet beverage of around 300 mL) [24]. Sucralose, on the other hand, is 600 times sweeter than sucrose and corresponds to a dose of 0.0558 g. The preloads were freshly prepared each morning of the test session by an uninvolved colleague to ensure blinding of the personnel conducting the study day and were administered at room temperature. The personnel involved in conducting the test sessions and blood analyses, as well as the participants, were blinded regarding the content of the preloads.

Fifteen minutes (*t* = 0 min) after the administration of the preload, a standard solid test meal was consumed, and *ad libitum* energy intake was measured. Blood samples (for CCK response and glycemic control) were collected and appetite-related sensations were assessed at *t* = −1, 15, 30, 60, 90, 120, 150, and 180 min. Appetite-related sensations were recorded using visual analogue scales (VASs) [28,29]. At *t* = −10 min, subjects were asked to rate the perceived sweetness and liking of the preload and at t = 180 min, the perceived liking of the test meal with the Global Sensory Intensity Scale (GSIS) and Global Hedonic Intensity Scale (GHIS), respectively [30]. Vital signs (blood pressure, heart rate) were measured at the beginning and at the end of each study day.

### 2.4. Materials

Erythritol, sucrose, and sucralose were purchased from regional suppliers (erythritol, Schweizer Edelzucker AG, St. Gallen, Switzerland; sucrose, Hänseler AG, Herisau, Switzerland; sucralose, Sigma-Aldrich, Buchs, Switzerland).

### 2.5. Composition and Conduction of the Test Meal

The test meal was freshly prepared every morning of the test session by the study personnel and consisted of ham sandwiches (78.5 g, 233.6 kcal per sandwich), cups of chocolate cream (50 g, 64.5 kcal per cup), and glasses of water and cooled orange juice (250 mL, 100 kcal per bottle). Each ham sandwich consisted of two slices of toast (56 g, 145.6 kcal), butter (10 g, 74.2 kcal), and one slice of ham (12.5 g, 13.8 kcal) and was cut to make four sandwich squares (19.6 g, 58.4 kcal per sandwich square). The chocolate cream, butter, ham, and orange juice were stored in the fridge at 7 °C. Participants were asked to eat and drink as much as they wanted but not for more than 20 min. However, the test meal ended as soon as the participant had stopped eating and/or drinking for more than 5 min because of maximum satiation. At irregular time intervals, food and drinks were served and refilled in excess to reduce the participant’s awareness of the amount of food consumed.

### 2.6. Assessment of Energy Intake

To assess the energy intake, the number of sandwich squares and chocolate cream cups consumed were recorded, and the volume (mL) of water and orange juice was measured before and after the test meal. Afterwards, the (total) energy intake was calculated.

### 2.7. Blood Sample Collection and Processing

Blood samples for the analysis of CCK were collected on ice into tubes containing ethylenediaminetetraacetic acid (EDTA) (6 µmol/L blood), a protease-inhibitor cocktail (Complete, EDTA-free, 1 tablet/50 mL blood, Roche, Mannheim, Germany), and a dipeptidyl peptidase IV inhibitor (10 µL/mL blood, Millipore Corp., St. Charles, MO, USA). Blood samples for the analysis of glucose and insulin were collected on ice into tubes containing EDTA (6 µmol/L blood) and a protease-inhibitor cocktail (Complete, EDTA-free, 1 tablet/50 mL blood, Roche, Mannheim, Germany). After centrifugation (4 °C, g force 1409, 10 min), plasma samples were immediately processed into different aliquots and stored at −80 °C until analysis.

### 2.8. Laboratory Analysis

Plasma CCK was measured with a sensitive radioimmunoassay using a highly specific antiserum (No. 92128) [31] (intra- and inter-assay variability below 15%; range of assay, 0.1 to 20 pmol/L). Plasma glucose was measured by a glucose oxidase method (Rothen Medizinische Laboratorien AG, Basel, Switzerland; range of assay, 0.6 to 45.0 mmol/L). Plasma insulin was quantified using a chemiluminescent microparticle immunoassay (chemiflex reagent kit (#8k41; Abbott), the relative light units detected by the ARCHITECT optical system (model: CI4100; Abbott), assay precision below 7% total CV; range of assay, 1.0 to 300.0 μU/mL).

### 2.9. Statistical Analysis

Data on a pure oral erythritol preload were not available. Based on a medium effect size (f = 0.31) for the difference in *ad libitum* energy intake after a sucrose versus sucralose preload [32], we determined that *n* = 20 yields 83% power to detect a similar difference. This sample size yields 80% power to detect a small effect size (f = 0.22) in the omnibus test of the mixed ANOVA comparing *ad libitum* energy intake after each of the four preloads, and 80% power to detect a medium effect size (d = 0.73) for the paired *t*-tests testing the specific hypotheses that erythritol will lead to a similar subsequent energy intake during an *ad libitum* test meal as sucrose and to a lower energy intake compared to sucralose and tap water, respectively, with multiple testing correction.

Data were analyzed in SAS 9.4 (SAS Institute, Cary, NC, USA) and reported as mean ± standard deviation (SD); the significance level was set at <0.05. Cohen’s d_z_ for paired *t*-tests was presented for effect sizes. For all analyses, the natural log-transformations of the dependent variables were used to normalize the distribution if the assumption of normally distributed residuals was violated (based on a significant *p*-value of the Shapiro–Wilk test). The visit number was included in all models to control for putative order effects. All outcome variables were analyzed using (generalized) linear mixed models on absolute values (energy intake, sweetness, and liking) or changes from baseline (CCK, glycemic control, and appetite-related sensations). “Preload” (energy intake, sweetness, and liking) and “time” (CCK, glycemic control, and appetite-related sensations) were included as within-subject independent variables in the models (including their main effects and the interaction). All models for CCK, glycemic control, and appetite-related sensations were controlled for the total energy intake. Planned contrast analyses were performed to test our specific hypotheses using Student’s t-tests with Tukey (for energy intake, sweetness, and liking) and stepdown Bonferroni–Holm correction for multiple testing (for CCK, glycemic control, and appetite-related sensations):

− *Comparison of energy intake between erythritol and sucrose, sucralose, or tap water* to test the hypothesis that erythritol will lead to a similar subsequent energy intake as sucrose and to a lower energy intake compared to sucralose or tap water.
− *Comparison of post-preload administration time point −1 min versus baseline values for each substance* to test the hypotheses that: (i) CCK will be released in response to erythritol and sucrose, but not in response to sucralose or tap water, (ii) the glucose and insulin concentrations will be increased in response to sucrose, but not in response to erythritol, sucralose, or tap water, and (iii) hunger/prospective food consumption will be decreased and satiety/fullness will be increased in response to erythritol and sucrose, but not in response to sucralose or tap water.
− *Comparison of post-preload administration time point −1 min versus baseline values between erythritol and sucrose, sucralose, or tap water* to test the hypotheses that: (i) CCK in response to erythritol will be similar to sucrose, but higher compared to sucralose or tap water, (ii) glucose and insulin concentrations will be lower in response to erythritol compared to sucrose, but similar between erythritol and sucralose or tap water, and (iii) hunger/prospective food consumption and satiety/fullness, respectively, in response to erythritol will be similar to sucrose, but lower and higher, respectively, compared to sucralose or tap water.
− *Comparison of post-preload administration time point 15 min (during the ad libitum test meal) versus baseline values between erythritol, sucrose, sucralose, or tap water* to explore CCK, glycemic control, and appetite-related sensations. No hypotheses were formulated beforehand.
− *Comparison of perceived sweetness and liking of the preloads, and perceived liking of the test meal between erythritol and sucrose, sucralose, or tap water* to test the hypothesis that erythritol will have a similar perceived sweetness as sucrose and sucralose, but higher compared to tap water. No differences will be observed in the perceived liking of the preloads and test meal between erythritol compared to sucrose, sucralose, or tap water.

To explore putative associations between CCK and energy intake, the differences between CCK concentrations between erythritol, sucrose, sucralose, or tap water at post-preload administration time point −1 min were correlated to the respective difference in energy intake by non-parametric Spearman’s correlation coefficient, Ρ.

## 3. Results

**Figure 1:**
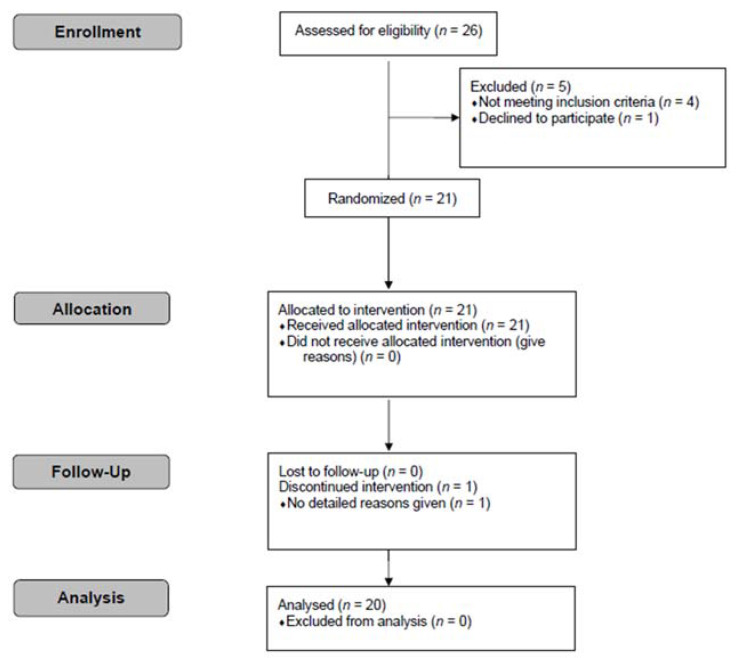
CONSORT flow diagram.

Twenty-one participants were allocated to the intervention. One dropped out due to personal reasons. Therefore, 20 participants (10 males and 10 females; mean ± SD (range), age: 29.3 ± 10.9 (21−54) years, BMI: 22.3 ± 1.6 (19.6–24.8) kg/m^2^) completed the study and complete data from 20 participants were available for analysis (Figure 1). All preloads were well tolerated.

### 3.1. Energy Intake and Total Energy Intake

**Figure 2:**
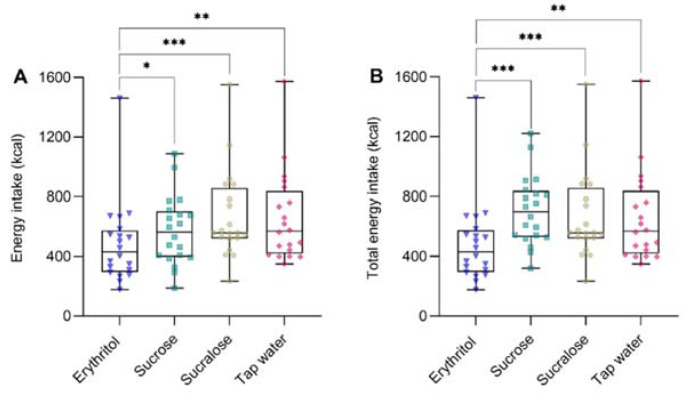
(**A**) Energy intake (kcal) from the *ad libitum* test meal and (**B**) total energy intake (kcal) (preload + *ad libitum* test meal) after oral administration of preloads containing either 50 g erythritol, 33.5 g sucrose, 0.0558 g sucralose, or tap water. Data are shown as median and interquartile range with individual values for each substance (triangle = erythritol, square = sucrose, circle = sucralose, and rhombus = tap water), and absolute values are presented. Statistics: linear mixed models followed by planned contrasts using post-hoc Student’s *t*-tests with Tukey correction for multiple testing in case of overall significance. *** *p_Tukey_* < 0.001; ** *p_Tukey_* < 0.01; * *p_Tukey_* < 0.05. *n* = 20.

A significant main effect of preload was found for the energy intake (F (3, 19) = 8.10, *p* = 0.001) and total energy intake (F (3, 19) = 16.67, *p* < 0.001). Planned contrast analyses showed that energy intake and total energy intake were lower after oral erythritol compared to sucrose, sucralose, or tap water (for all comparisons, *p_Tukey_* < 0.05 and d_z_ ≥ 0.68). Figure 2 and Table 1 show the energy intake from the *ad libitum* test meal and the total energy intake (preload and *ad libitum* test meal).

### 3.2. GI Satiation Hormone: Plasma CCK

**Figure 3:**
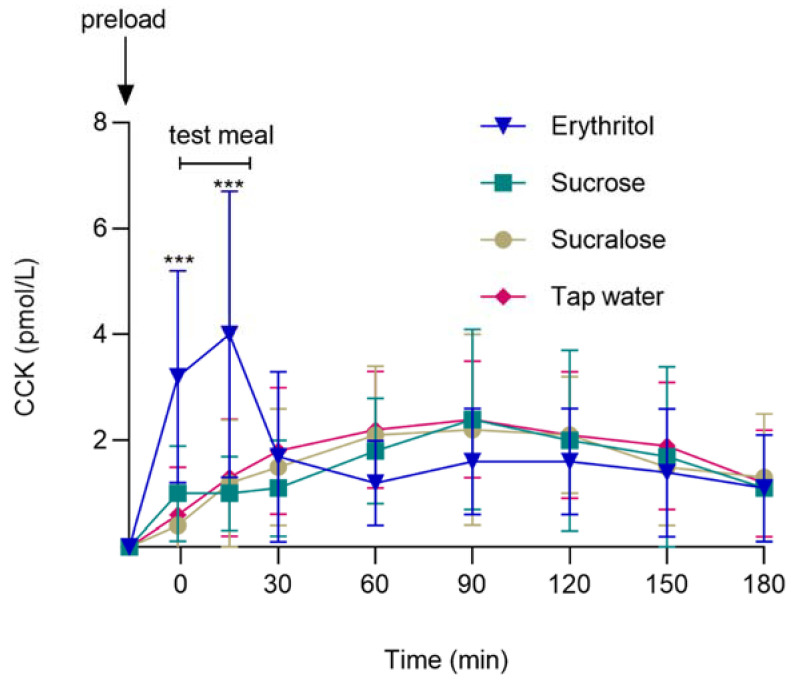
CCK concentrations after oral administration of either 50 g erythritol, 33.5 g sucrose, 0.0558 g sucralose, or tap water, and after the ad libitum test meal. Data are shown as mean ± SD, and baseline values are presented. Statistics: linear mixed models followed by planned contrasts with Holm correction for multiple testing. *** *p_Holm_* < 0.001 erythritol vs. sucrose, sucralose, and tap water. CCK, cholecystokinin. *n* = 20.

The main effect of preload was significant for CCK (F (3, 64) = 3.99, *p* = 0.011). Furthermore, the preload-by-time interaction effect was significant for CCK (F (21, 290) = 5.76, *p* < 0.001). Erythritol and sucrose induced a significant increase in CCK, whereas sucralose and tap water had no effect before the start of the *ad libitum* test meal. Planned contrast analyses showed that CCK responses were higher after oral erythritol compared to sucrose, sucralose, or tap water at −1 min (before the start of the *ad libitum* test meal) and at 15 min (during the *ad libitum* test meal) (for all: comparisons of the changes from baseline, all *p_Holm_ <* 0.001, d_z_ ≥ 1.51). Figure 3 and Table 2 show the CCK secretion in response to oral erythritol, sucrose, sucralose, or tap water.

### 3.3. Associations between CCK and Energy Intake

The difference in CCK concentrations between oral erythritol and sucrose, sucralose, or tap water were not associated with the respective difference in energy intake (P = −0.212, P = −0.234, P = 0.053, respectively, all *p* > 0.05).

### 3.4. Glycemic Control: Plasma Glucose and Insulin

**Figure 4:**
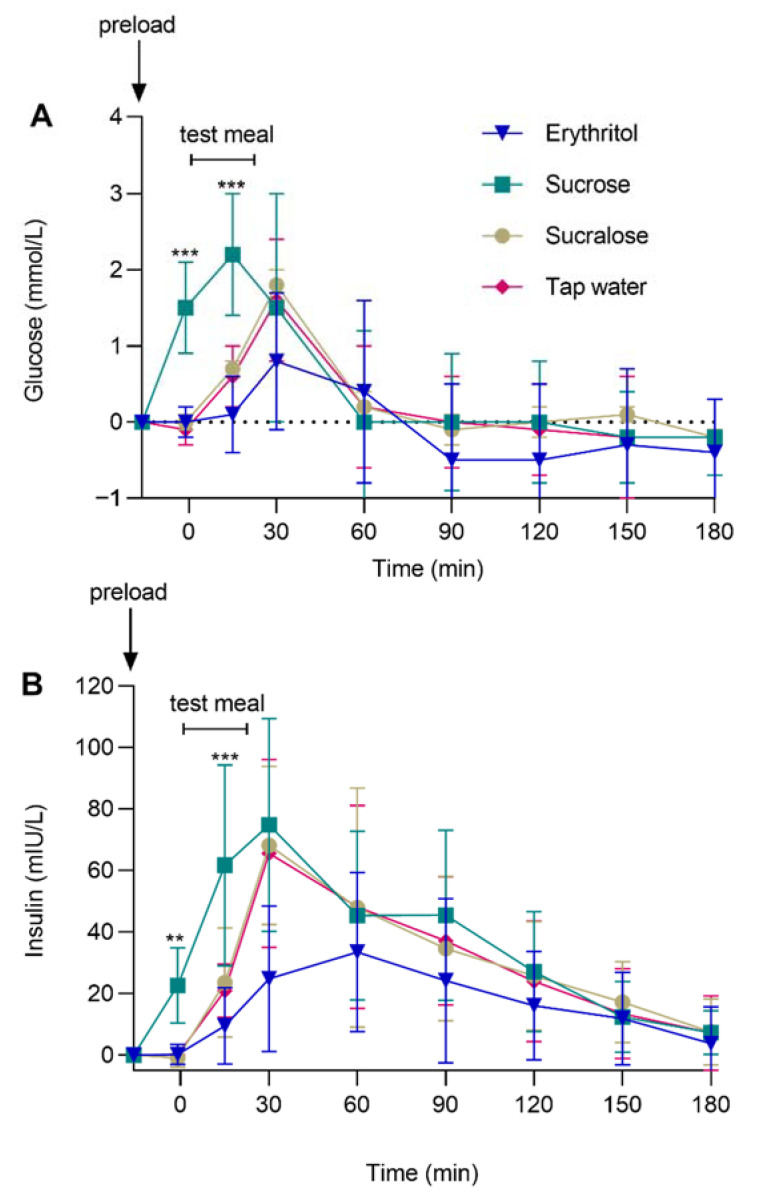
(**A**) Glucose and (**B**) insulin concentrations after oral administration of preloads containing either 50 g erythritol, 33.5 g sucrose, 0.0558 g sucralose, or tap water, and after the ad libitum test meal. Data are shown as mean ± SD, and baseline values are presented. Statistics: linear mixed models followed by planned contrasts with Holm correction for multiple testing. *** *p_Holm_* < 0.001 and ** *p_Holm_* < 0.01 for erythritol vs. sucrose. *n* = 20.

The main effect of preload was significant for glucose (F (3, 74) = 4.98, *p* = 0.003) and insulin (F (3, 70) = 8.89, *p* < 0.001). Furthermore, the preload-by-time interaction effect was significant for glucose (F (21, 290) = 5.79, *p* < 0.001) and insulin (F (21, 288) = 6.29, *p* < 0.001). Sucrose induced a significant increase in glucose and insulin concentrations, whereas erythritol, sucralose, and tap water had no effect before the start of the ad libitum test meal. Planned contrast analyses showed that glucose and insulin responses were lower after oral erythritol than after sucrose (for both: comparisons of the changes from baseline, *p_Holm_* < 0.01, d_z_ ≥ 0.87), with no difference between erythritol and sucralose or tap water at −1 min (before the start of the ad libitum test meal) and at 15 min (during the ad libitum test meal) (for all: comparisons of the changes from baseline, all *p_Holm_* > 0.05). Figure 4 and Table 2 show glucose and insulin concentrations in response to oral erythritol, sucrose, sucralose, or tap water.

### 3.5. Appetite-Related Sensations: Hunger, Prospective Food Consumption, Satiety, and Fullness

#### 3.5.1. Hunger

Neither the main effect of preload (F (3, 62) = 2.13, *p* = 0.106) nor the preload-by-time interaction effect (F (21, 288) = 0.96, *p* = 0.520) were significant. Planned contrast analyses showed that hunger was lower after oral erythritol compared to tap water at −1 min (before the start of the ad libitum test meal, *p_Holm_* = 0.003, d_z_ = 0.77), but not at 15 min (during the ad libitum test meal, *p_Holm_* = 0.094). There was no difference between erythritol and sucrose or sucralose at −1 and 15 min (for all: comparisons of the changes from baseline, all *p_Holm_* > 0.05).

#### 3.5.2. Prospective Food Consumption, Satiety, and Fullness

Neither the main effects of preload ((F (3, 62) = 0.27, *p* = 0.848), (F (3, 59) = 0.25, *p* = 0.862), and (F (3, 58) = 0.25, *p* = 0.874), respectively) nor the preload-by-time interaction effects ((F (21, 290) = 1.34, *p* = 0.205), (F (21, 288) = 0.61, *p* = 0.912), and (F (21, 288) = 1.35, *p* = 0.140), respectively) were significant. None of the planned contrast analyses were significant. Table 2 shows appetite-related sensations in response to oral erythritol, sucrose, sucralose, or tap water.

#### 3.5.3. Perceived Sweetness and Liking of the Preloads

A significant main effect of preload was found for the perceived sweetness of the preloads (F (3, 19) = 77.43, *p* < 0.001). Planned contrast analyses showed that the perceived sweetness of the preload was not different between oral erythritol and sucrose (*p_Tukey_* = 0.665), but higher after erythritol compared to sucralose and tap water (*p_Tukey_* = 0.002 and *p_Tukey_* < 0.001, respectively). No significant main effect of preload was found for the perceived liking of the preloads (F (3, 19) = 1.30, *p* = 0.304). None of the planned contrast analyses were significant.

#### 3.5.4. Perceived Liking of the Test Meal

No significant main effect of preload was found for perceived liking of the test meal (F (3, 19) = 1.45, *p* = 0.260). None of the planned contrast analyses were significant.

## 4. Discussion

In this double-blinded, four-way crossover study in healthy participants, the effects of oral erythritol on energy intake compared to sucrose, sucralose, or tap water during a subsequent *ad libitum* test meal were investigated. The results can be summarized as follows: (1) The energy intake from the *ad libitum* test meal and the total energy intake (preload + *ad libitum* test meal) were significantly lower after erythritol compared to sucrose, sucralose, or tap water. (2) Erythritol led to a significant increase in CCK compared to sucrose, sucralose, or tap water before the start of the *ad libitum* test meal. (3) Glucose and insulin concentrations were significantly lower after erythritol compared to sucrose with no significant difference between erythritol and sucralose or tap water.

The role of artificial LCS and their impact on obesity and T2DM is highly debated; the alternatives, such as low-caloric bulk sweeteners (e.g., erythritol), are more intensely researched. Overduin et al. [27] partially replaced sucrose by erythritol in a test breakfast and reported that the energy intake during the subsequent *ad libitum* test meal was similar between the two breakfasts (sucrose or sucrose + erythritol). Moreover, the release of GLP-1 and PYY including appetite-related sensations were comparable between the sucrose and sucrose + erythritol test breakfast [27]. Our results are different as we show a significantly reduced energy intake after the oral intake of erythritol alone compared to sucrose suggesting that the satiation effect of erythritol is greater than that of sucrose.

Artificial LCS are frequently used in foods and beverages. In a recent meta-analysis, which included several human studies, Lee et al. [33] analyzed the effects of unsweetened preloads and preloads sweetened with either LCS or caloric sugars on subsequent energy intake. The total energy intake after unsweetened preloads or after preloads sweetened with LCS followed by an *ad libitum* test meal was lower compared to preloads sweetened with caloric sugars. Of note, the energy intake without the calories of the preloads was greater for the unsweetened and LCS-sweetened preloads compared to the preloads with caloric sugars, with no significant differences between the unsweetened and LCS-sweetened preloads. The authors conclude that the caloric differences of the preloads rather than differences in sweetness account for the results. The preloads with caloric sugars possibly resulted in a satiation effect during the *ad libitum* test meal [33,34]. The meta-analysis included studies with different designs: (1) various LCS were included, including artificial and bulk LCS and caloric sugars, and (2) the test meal composition and the time between preload and test meal were variable. All these factors can influence individual effects on energy intake. It is therefore interesting to note that the results indicate similar trends as found in the present study, although not formally statistically tested, as follows: the energy intake after caloric preloads is decreased compared to LCS-sweetened (e.g., sucralose) or unsweetened preloads (water) but the total energy intake is greater due to the calories of the preload. These studies highlight a notable difference between the artificial LCS sucralose and the natural bulk sweetener erythritol. Artificial LCS and water were not able to induce satiation and reduce energy intake during *ad libitum* test meals [33]. Erythritol, on the other hand, seems to induce a satiation effect comparable to that of sucrose as shown by Overduin et al. [27] and indicated in the present study.

A possible explanation for the differences in energy intake between erythritol and sucrose or sucralose might be the secretion of GI satiation hormones. In the present study, oral erythritol resulted in a strong CCK release until time point 15 min during the *ad libitum* test meal. These results are in line with previous studies [23,24,25] and might partially explain the reduced energy intake. A recent review reported that CCK and its analogues have a significant effect on satiation [35]. In fact, not only CCK but also GLP-1 and PYY are linked to a reduced energy intake and, in addition, with a delay in gastric emptying [36,37,38,39]. For erythritol, a reduction in gastric emptying has previously been observed [24,25] and seems to contribute to a reduced energy intake. Sucrose can both stimulate GLP-1 and PYY release [27,40,41] and induce a delayed gastric emptying in humans [42]. In this trial, we only observed a small release of CCK in response to sucrose compared to erythritol before the start of the *ad libitum* test meal. In the present study, sucrose also affected satiation because the subsequent energy intake was lower compared to sucralose and tap water. In contrast, sucralose does not stimulate the release of GI satiation hormones in humans [6,7,9,43] as confirmed in the present study. The observation is in line with the meta-analysis by Lee et al. [33]. The detailed mechanisms of the GI satiation hormone secretion (especially CCK and GLP-1) in response to erythritol are still unknown. One hypothesis was the stimulation of GLP-1 secretion via the activation of the sweet taste receptor located on enteroendocrine cells (EECs) in the gut as previously shown for glucose [44]. However, inhibiting the sweet taste receptor did not affect the erythritol-stimulated GLP-1 release [25]. Another possible mechanism involves the sodium-glucose transporter-1 (SGLT-1) for glucose-induced GLP-1 release [45,46,47]. However, studies to date with erythritol and SGLT-1 are lacking. In addition, at least in mice, GLP-1 induced satiation requires vagal CCK receptor activation [48].

Another possible explanation for the difference in energy intake between erythritol and sucralose might be the differences in neuroepithelial circuits. A previous hypothesis suggested that the brain largely senses nutrients via the passive release of GI hormones [49]. However, Bohórquez et al. [50] found a neuroepithelial circuit where EECs synapse with vagal neurons. This gut-brain circuit enables the transduction of sugar signals in milliseconds by using the neurotransmitter glutamate [51]. They call this EEC innervation neuropod cells [50]. Recently, the same research group reported that the preference for sucrose over sucralose in mice depends on duodenal neuropod cells [52]. These neuropod cells convey signals to the vagus nerve by using two individual neural pathways. While sucralose activates the sweet taste receptor subunit T1R3 to promote the release of adenosine triphosphate (ATP), sucrose enters the neuropod cell via the SGLT-1 and stimulates the release of glutamate [52]. Thus, to discern sucrose from sucralose, glutamatergic signaling is necessary [52]. Whether these mechanisms apply to guide nutritive choices and have an impact on subsequent energy intake in humans remains to be determined. Therefore, more research is required to investigate whether these neuroepithelial mechanisms are applicable to erythritol and transferable to humans.

In the current study, glucose and insulin concentrations were affected neither after oral erythritol nor after sucralose intake before the *ad libitum* test meal, supporting results in previous studies [23,53]. Additionally, the oral intake of erythritol over 7 weeks had no effect on glycemic control (unpublished, Meyer-Gerspach et al., 2018), nor on glucose absorption [54]. However, when sucralose was administered together with carbohydrates (typical scenario in a real-world setting), insulin sensitivity was decreased in healthy humans [17,55]. An upregulation of SGLT-1 and glucose transporter 2 (GLUT2) (as observed in mice) might be an explanation, which results in an increased glucose absorption [18,19]. The hypothesis has yet to be tested in humans.

The strengths of our study comprise the study design (randomized, controlled, double-blinded, cross-over design), which reduces interindividual variability as well as the comparison of erythritol to one of the most widely used sugars, sucrose, and the artificial LCS, sucralose. Some limitations of the study require consideration. First, only acute effects of preloads on subsequent energy intake were investigated. The effect of chronic exposure needs to be investigated. Hence, the results cannot be extrapolated to chronic intake. Second, the intake of a preload in the form of a liquid drink and not in the form of a solid snack can influence satiation due to different effects on the cephalic phase of ingestion. Third, the comparison to other energy intake studies is difficult since differences in design, such as the time points between preloads and test meal intake as well as their compositions, have major impacts on satiation and energy intake. Nonetheless, the results are relevant because they show novel insights into two sweeteners and their effects on energy intake representing an every-day scenario.

In conclusion, we show that oral erythritol given before an *ad libitum* meal induces the release of the GI satiation hormone CCK and reduces subsequent energy intake compared to sucrose, sucralose, or tap water. Moreover, erythritol has no effect on glucose and insulin concentrations supporting a role as a useful sugar alternative.

## Figures and Tables

**Table 1:** Effects of preloads containing either (A) 50 g erythritol, (B) 33.5 g sucrose, (C) 0.0058 g sucralose, or (D) tap water on energy intake (*ad libitum* test meal) and total energy intake (preload + *ad libitum* test meal) in 20 healthy participants ^1^.

Parameter.	Erythritol *n* = 20	Sucrose *n* = 20	Sucralose *n* = 20	Tap Water *n* = 20	*p*-Value (Overall)	*p*-Values (Post Hoc)	Effect Size
**Energy intake** **(kcal)**	483 ± 277	573 ± 230	669 ± 297	655 ± 300	*p* = 0.001	A vs. B: *p* = 0.030 A vs. C: *p* < 0.001 A vs. D: *p* = 0.003	d_z_ = 0.68 d_z_ = 1.08 d_z_ = 0.93
**Total energy intake (kcal)**	483 ± 277	707 ± 230	669 ± 297	655 ± 300	*p* < 0.001	A vs. B: *p* < 0.001 A vs. C: *p* < 0.001 A vs. D: *p* = 0.003	d_z_ = 1.49 d_z_ = 1.08 d_z_ = 0.93

^1^ Data are shown as mean ± SD and presented as absolute values. Statistics: linear mixed models followed by planned contrasts using post-hoc Student’s *t*-tests with Tukey correction for multiple testing in case of overall significance and Cohen’s d_z_ for paired *t*-tests (effect sizes); d_z,_ the effect size estimate.

**Table 2:** Effects of preloads containing either 50 g erythritol, 33.5 g sucrose, 0.0058 g sucralose, or tap water on CCK, glycemic control, and appetite-related sensations in 20 healthy participants ^1^.

Parameters	Time Points	Preloads			*p*-Values	
		Erythritol vs. Sucrose	Erythritol vs. Sucralose	Erythritol vs. Tap Water	Main Effect of Preload	Preload-by-Time Interaction
CCK (pmol/L)	−1 min	0.43 ± 0.06	0.62 ± 0.07	0.52 ± 0.07	*p* = 0.011	*p* < 0.001
		*p_Holm_* < 0.001	*p_Holm_* < 0.001	*p_Holm_* < 0.001		
		d_z_ = 1.51	d_z_ = 1.89	d_z_ = 1.71		
	15 min	0.07 ± 0.01	0.07 ± 0.01	0.06 ± 0.01		
		*p_Holm_* < 0.001	*p_Holm_* < 0.001	*p_Holm_* < 0.001		
		d_z_ = 1.76	d_z_ = 1.46	d_z_ = 1.43		
Glucose (mmol/L)	−1 min	−0.92 ± 0.18	0.06 ± 0.16	0.09 ± 0.18	*p* = 0.003	*p* < 0.001
		*p_Holm_* < 0.001	*p_Holm_* = 1	*p_Holm_* = 1		
		d_z_ = 1.16				
	15 min	−0.18 ± 0.03	−0.05 ± 0.02	−0.04 ± 0.02		
		*p_Holm_* < 0.001	*p_Holm_* = 0.053	*p_Holm_ =* 0.085		
		d_z_ = 1.61				
Insulin (mIU/L)	−1 min	−0.99 ± 0.25	0.33 ± 0.24	0.22 ± 0.23	*p* < 0.001	*p* < 0.001
		*p_Holm_* < 0.01	*p_Holm_* = 0.344	*p_Holm_* = 0.344		
		d_z_ = 0.87				
	15 min	−0.28 ± 0.04	−0.07 ± 0.03	−0.07 ± 0.03		
		*p_Holm_* < 0.001	*p_Holm_* = 0.074	*p_Holm_* = 0.074		
		d_z_ = 1.73				
Hunger (cm)	−1 min	−0.66 ± 0.30	−0.32 ± 0.26	−0.94 ± 0.27	*p* = 0.106	*p* = 0.520
		*p_Holm_* = 0.065	*p_Holm_* = 0.210	*p_Holm_* = 0.003		
				d_z_ = 0.77		
	15 min	−0.09 ± 0.04	−0.04 ± 0.04	−0.08 ± 0.04		
		*p_Holm_ =* 0.094	*p_Holm_* = 0.257	*p_Holm_ =* 0.094		
Pfc (cm)	−1 min	−0.05 ± 0.33	−0.14 ± 0.30	−0.38 ± 0.29	*p* = 0.848	*p* = 0.205
		*p_Holm_* = 1	*p_Holm_* = 1	*p_Holm_* = 0.558		
	15 min	−0.02 ± 0.05	−0.05 ± 0.04	−0.02 ± 0.04		
		*p_Holm_* = 1	*p_Holm_* = 0.725	*p_Holm_* = 1		
Satiety (cm)	−1 min	0.03 ± 0.43	0.08 ± 0.38	0.34 ± 0.40	*p* = 0.862	*p* = 0.912
		*p_Holm_* = 1	*p_Holm_* = 1	*p_Holm_* = 1		
	15 min	0.02 ± 0.06	0.05 ± 0.05	0.02 ± 0.06		
		*p_Holm_* = 1	*p_Holm_* = 1	*p_Holm_* = 1		
Fullness (cm)	−1 min	0.19 ± 0.29	0.19 ± 0.29	0.52 ± 0.28	*p* = 0.874	*p* = 0.140
		*p_Holm_* = 1	*p_Holm_* = 1	*p_Holm_* = 0.190		
	15 min	0.03 ± 0.04	0.03 ± 0.04	0.01 ± 0.04		
		*p_Holm_* = 1	*p_Holm_* = 1	*p_Holm_* = 1		

^1^ Estimates from linear mixed models are shown as means ± standard error and present the changes from baseline for erythritol vs. sucrose, sucralose, or tap water at −1 min and at 15 min. Statistics: linear mixed models followed by planned contrasts with Holm correction for multiple testing and Cohen’s d_z_ for paired *t*-tests (effect sizes). CCK, cholecystokinin; pfc, prospective food consumption.

## Data Availability

Data described in the manuscript and code book will be made publicly and freely available without restriction at https://github.com/labgas/proj_erythritol_5.
